# Diagnosis and management of dilated cardiomyopathy: a systematic review of clinical practice guidelines and recommendations

**DOI:** 10.1093/ehjqcco/qcae109

**Published:** 2024-12-14

**Authors:** Anna Sorella, Kristian Galanti, Lorena Iezzi, Sabina Gallina, Selma F Mohammed, Neha Sekhri, Mohammed Majid Akhtar, Sanjay K Prasad, Choudhary Anwar Ahmed Chahal, Fabrizio Ricci, Mohammed Yunus Khanji

**Affiliations:** Department of Neuroscience, Imaging and Clinical Sciences, G. D'Annunzio University of Chieti-Pescara, 66100 Chieti, Italy; Department of Neuroscience, Imaging and Clinical Sciences, G. D'Annunzio University of Chieti-Pescara, 66100 Chieti, Italy; Department of Neuroscience, Imaging and Clinical Sciences, G. D'Annunzio University of Chieti-Pescara, 66100 Chieti, Italy; Department of Neuroscience, Imaging and Clinical Sciences, G. D'Annunzio University of Chieti-Pescara, 66100 Chieti, Italy; University Cardiology Division, Heart Department, SS. Annunziata Polyclinic, Chieti 66100, Italy; Institute for Advanced Biomedical Technologies, G. D'Annunzio University of Chieti-Pescara, 66100 Chieti, Italy; Department of Cardiology, Creighton University School of Medicine, Omaha, NE 68124, USA; Barts Heart Centre, St. Bartholomew's Hospital, Barts Health NHS Trust, West Smithfield, London EC1A 7BE, UK; Newham University Hospital, Barts Health NHS Trust, London EC1M 6BQ, UK; William Harvey Research Institute, NIHR Barts Biomedical Centre, Queen Mary University of London, Charterhouse Square, London EC1M 6BQ, UK; Royal Brompton and Harefield Hospitals, Guy's and St Thomas’ NHS Foundation Trust, London SW3 6NP, UK; Royal Brompton and Harefield Hospitals, Guy's and St Thomas’ NHS Foundation Trust, London SW3 6NP, UK; National Heart and Lung Institute, Imperial College London, London SW7 2AZ, UK; Barts Heart Centre, St. Bartholomew's Hospital, Barts Health NHS Trust, West Smithfield, London EC1A 7BE, UK; William Harvey Research Institute, NIHR Barts Biomedical Centre, Queen Mary University of London, Charterhouse Square, London EC1M 6BQ, UK; Center for Inherited Cardiovascular Diseases, Department of Cardiology, WellSpan Health, 30 Monument Rd, York, PA 17403, USA; Department of Cardiovascular Medicine, Mayo Clinic, 200 First Str, SW Rochester, MN 55905, USA; Department of Neuroscience, Imaging and Clinical Sciences, G. D'Annunzio University of Chieti-Pescara, 66100 Chieti, Italy; University Cardiology Division, Heart Department, SS. Annunziata Polyclinic, Chieti 66100, Italy; Institute for Advanced Biomedical Technologies, G. D'Annunzio University of Chieti-Pescara, 66100 Chieti, Italy; Barts Heart Centre, St. Bartholomew's Hospital, Barts Health NHS Trust, West Smithfield, London EC1A 7BE, UK; Newham University Hospital, Barts Health NHS Trust, London EC1M 6BQ, UK; William Harvey Research Institute, NIHR Barts Biomedical Centre, Queen Mary University of London, Charterhouse Square, London EC1M 6BQ, UK

**Keywords:** Dilated cardiomyopathy, Multimodality cardiovascular imaging, Risk stratification, Guidelines recommendations, Cardiogenetics

## Abstract

Dilated cardiomyopathy (DCM) is extensively discussed in numerous expert consensus documents and international guidelines, with differing recommendations. To support clinicians in daily practice and decision-making, we conducted a systematic review of key guidelines and recommendations concerning the diagnosis and clinical management of DCM. Our research encompassed MEDLINE and EMBASE databases for relevant articles published, as well as the websites of relevant scientific societies. We identified two guidelines and one scientific statement that met stringent criteria, thereby qualifying them for detailed systematic analysis. Our review revealed consensus on several key aspects: the definition of DCM, the use of B-type natriuretic peptides and high-sensitivity troponin in laboratory testing, the essential role of multimodality cardiovascular imaging for initial diagnosis, genetic counselling, and the management of advanced disease. Nonetheless, notable areas of variation included the formation of multidisciplinary management teams, the role of cascade genetic testing, pathways for arrhythmic risk stratification, and the criteria for prophylactic defibrillator implantation. Significant evidence gaps persist, particularly regarding the clinical trajectory of genetic, non-genetic and gene-elusive forms of DCM, the use of cardiovascular magnetic resonance in phenotype-negative family members with genotype-positive probands, and the development of potential aetiology-oriented therapies. Addressing these gaps could enhance clinical outcomes and inform future research directions and guideline development.

**Abbreviations**
^18^F-FDG-PET: ^18^F-fluorodeoxyglucose positron emission tomography
ACC: American College of Cardiology
AGREE: Appraisal Guidelines for Research and Evaluation
AHA: American Heart Association
ANP: atrial natriuretic peptide
BNP: B-type natriuretic peptide
CAD: coronary artery disease
CCTA: coronary computed tomography angiography
CMR: cardiovascular magnetic resonance
CT: computed tomography
DCM: dilated cardiomyopathy
DSP: desmoplakin
EF: ejection fraction
ESC: European Society of Cardiology
FLNC: filamin C
GDMT: guideline-directed medical therapy
I-123-BMIPP: 15-(4-^123^I-iodophenyl)-3(*R,S*)-methylpentadecanoic acid
I-123-MIBG: ^123^I-metaiodobenzylguanidine
ICD: implantable cardioverter defibrillator
JCS: Japanese Circulation Society
JHFS: Japanese Heart Failure Society
LMNA: lamin A/C
LV: left ventricular
LVAD: left ventricular assist device
MCS: mechanical circulatory support
MDT: multidisciplinary team
MYBPC3: myosin-binding protein C3 (isoform)
MYH7: myosin heavy chain beta 7 (isoform)
NCS: non-cardiac surgery
NSVT: non-sustained ventricular tachycardia
NT-proBNP: amino-terminal pro-B-type natriuretic peptide
NYHA: New York Heart Association
PLN: phospholamban
SCD: sudden cardiac death
TMEM43: transmembrane protein 43
TTE: transthoracic echocardiography
TTN: titin
VUS: variants of unknown significance

Key learning points **What is already known**

- Dilated cardiomyopathy (DCM) is extensively covered in existing scientific literature, but dedicated guidelines specifically addressing this condition are lacking. Much of the clinical guidance for DCM overlaps with heart failure management, reflecting the shared aspects of treatment strategies. However, specific nuances and targeted recommendations for DCM remain less emphasized in broader clinical practice guidelines.

**What this study adds**

- This systematic review of clinical practice guidelines and other recommendations on the diagnosis and management of DCM highlights areas of consensus, disagreement, and current gaps in the evidence or guidelines. This document is intended to serve as a valuable resource for supporting clinicians in the management of DCM patients and to guide future research efforts.

## Introduction

Dilated cardiomyopathy (DCM) is defined as a heart muscle disease characterized by left ventricular (LV) dilatation and global or regional systolic dysfunction, not attributable to abnormal loading conditions (e.g. hypertension, valvular, or congenital heart disease) or coronary artery disease (CAD).^1^ Over recent decades, reclassifications and evolving definitions of DCM have introduced variability in data regarding epidemiology and clinical management.^2^ Recent reviews suggest that DCM prevalence may range from 1:250 to 1:400 individuals in the general population.^3^ Notably, familial DCM accounts for 30–50% of cases, with ∼30–40% of these attributable to identifiable genetic causes.^4–6^ The therapeutic management of DCM has traditionally overlapped with heart failure (HF) treatment, sharing primary therapeutic options, from optimal decongestion to appropriate risk stratification for arrhythmias. Advances in the field of cardiogenetics and the impact of deep phenotyping through advanced imaging with the capacity of tissue characterization have introduced a new diagnostic perspective and have contributed to the detection of subclinical forms of disease. It is now understood that DCM encompasses a wide spectrum of aetiologies, ranging from overt DCM to intermediate phenotypes within the DCM spectrum. These may present as early heart rhythm disorders without overt structural disease, isolated LV dilation, isolated scar, or hypokinetic non-dilated cardiomyopathy.^7^ Despite well-established genotype–phenotype correlations for specific gene variants [e.g. desmoplakin (DSP), filamin-C (FLNC), and laminin], myocardial resilience to disease progression in gene-related, familial gene elusive, acquired, and idiopathic forms remains largely unknown. For instance, the identification of DCM patients who are at risk of disease progression remains obscure, making it challenging to distinguish those who might benefit from early treatment and potentially experience improvement or remission.^8^ Therefore, our aim was to conduct a systematic review of current guidelines and recommendations from major scientific societies regarding the diagnosis and management of DCM. We sought to highlight areas of consensus and discrepancy, with the intention of guiding clinical management and informing future research initiatives.

## Methods

### Data sources and searches

We conducted a systematic review of English language international guidelines and scientific documents, concerning the diagnosis and management of DCM. Our research was conducted in April 2024 including MEDLINE and EMBASE, extending back to 2014. Additionally, we searched specific websites of leading international health organizations relevant to guideline development (see Supplementary material online, *Table S1*). This systematic review was planned and reported based on the Preferred Reporting Items for Systematic Reviews and Meta-Analyses (PRISMA) recommendations.^9^

### Study selection

The documents selected and included in this review were authored by international scientific societies that have issued specific recommendations for the diagnosis and management of DCM in adults. This systematic review encompasses both formal guidelines and relevant scientific documents that were deemed to be rigourously developed. In the case of the Japanese guidelines, it was necessary to include the 2021 update alongside the original 2018 document. For the other documents, no updated versions are currently available. The authors developed a search syntax that served as a basis for the search strategy (see Supplementary material online).

### Data extraction and quality assessment

Titles and abstracts were evaluated by two independent reviewers (A.S. and K.G.) using Rayyan.ai (https://www.rayyan.ai/). Articles were excluded if both reviewers agreed on their ineligibility. Any discrepancies were resolved through final discussion and consensus among the authors. Both reviewers conducted the final selection for complete data extraction. The 23-item Appraisal of Guidelines for Research and Evaluation (AGREE) II tool was used to determine the rigour of development for each guideline and/or scientific document. Two reviewers (A.S. and K.G.) independently scored each item according to the AGREE II instructions. The average rigour scores were calculated by expressing the sum of individual scores as a percentage of the maximum possible score. Editorial independence from the funding body was assessed, as well as external funding and disclosure of relationships with industry by individual guideline group members. The guidelines were ranked according to their scores. The reproducibility of reviewers’ scores was excellent, with an intraclass correlation of 0.96 (see Supplementary material online, *Table S2*).

### Data synthesis and analysis

Two reviewers (A.S. and K.G.) independently extracted all relevant recommendations from the guidelines and scientific documents that achieved an AGREE II score of ≥50% and compiled a comprehensive recommendation matrix.

## Results

**Figure 1:**
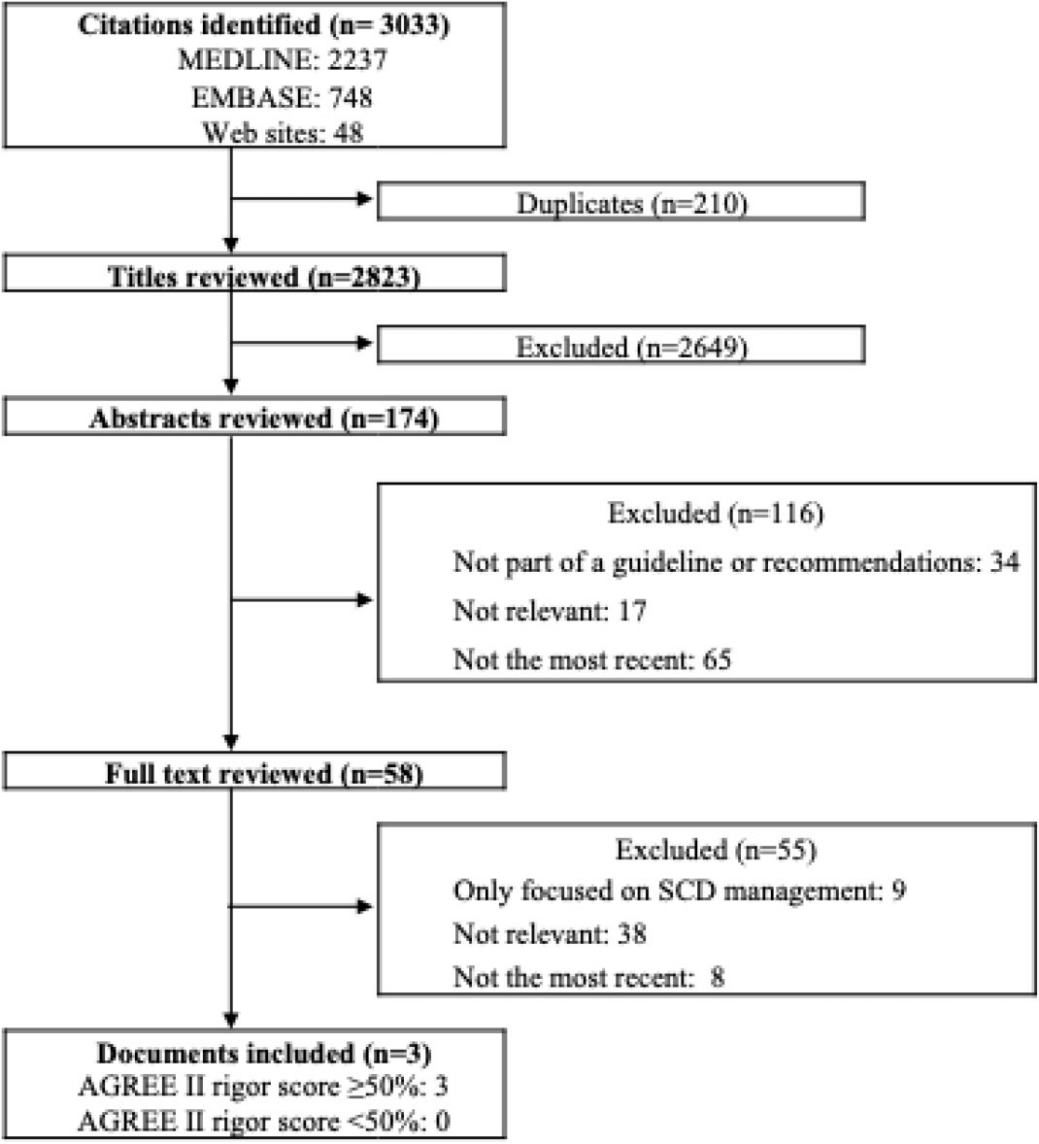
Summary of guideline search and review process. AGREE II, Appraisal of Guidelines for Research and Evaluation II.

We retrieved 3033 titles, of which 174 were deemed potentially eligible. Upon thorough review of all manuscripts, we selected two guidelines and one scientific statement pertinent to the diagnosis and management of DCM. The included documents were sourced from the following international societies: the American Heart Association/American College of Cardiology^10^ (AHA/ACC), the European Society of Cardiology^1^ (ESC), and the Japanese Circulation Society/Japanese Heart Failure Society^11^ (JCS/JHFS). *Table 1* summarizes the content of the three selected articles, highlighting the main topics and recommendations reviewed, the rigour scores, and any potential conflicts of interest. All documents, each achieving an AGREE II score exceeding 50%, constitute the core of our analysis (*Figure 1*). The inclusion of the AHA scientific statement in our systematic review was justified due to its comprehensive scope and reliance on multidisciplinary expertise. It integrates recommendations derived from established guidelines by the ACC/AHA and other organizations, offering in-depth insights into the classification, diagnosis, and management of DCM. Although incorporating the scientific statement may align with contemporary clinical practices in cardiomyopathy management, it should be highlighted that the development process may be less stringent than formal guidelines and may be prone to additional limitations and bias.

**Table 1:** Recommendations for dilated cardiomyopathy diagnosis and management

Organization society	European Society of Cardiology, ESC	American Heart Association, AHA	Japanese Circulation Society, JCS
Document type	Clinical practice guidelines	Scientific statement	Clinical practice guidelines
Region applied	Europe	USA	Japan
Year of publication	2023	2016	2018 (update 2021)
AGREE II rigour score, %	94%	97%	88%
Conflicts of interest	EI, SCI^a,b^	EI, SCI^a^	EI, SCI^a,b^
Methods used to evaluate evidence	Systematic review	Systematic review	Systematic review
Methods used to formulate recommendations	Formal consensus	Formal consensus	Formal consensus
Consideration of costs	Cost considered in strength of recommendation	Information from studies on cost considered where available	Cost considered in strength of recommendation
Definition of DCM	− LV dilatation + − Global or regional systolic dysfunction unexplained solely by abnormal loading conditions (e.g. hypertension, valve disease, and CHD) or CAD	− Ventricular dilation + − Depressed myocardial performance in the absence of hypertension, valvular, congenital or ischaemic heart disease	− Global LV dysfunction + − LV dilatation + − Exclusion of ‘secondary CMP’
Diagnostic workup	− Multiparametric approach for suspected or established CMP: clinical evaluation, pedigree analysis, ECG, Holter monitoring, laboratory tests and MMI (I-C) − Evaluation of family history and construction of a three-to-four-generation family pedigree (I-C)	− Multiparametric approach for unexplained CMP (patient with LVEF <50%): detailed history, three-generational family history, ECG, echocardiography, chest radiography, laboratory tests − Disease-specific recommendations	− Clinical evaluation, pedigree analysis, standard ECG, Holter ECG, exercise testing, laboratory tests, and multimodality imaging are considered in the diagnostic workup
Laboratory tests	− First-level tests for aetiology evaluation, disease severity assessment, and extracardiac manifestations: Ca^2+^, CK, ferritin, full blood count, liver function, NT-proBNP, phosphate, proteinuria, renal function, serum iron, thyroid function, troponin, and vitamin D (children) (I-C) − Second-level tests in patients with extracardiac features for metabolic and syndromic aetiologies: carnitine profile, FFAs, organ- and non-organ-specific serum autoantibodies, ACE, thiamine, viral serology, urine organic acids, and plasma amino acids (IIa-C)	− Complete blood count − BNP and/or NT-proBNP − Troponin − Metabolic panel: serum electrolytes including Ca^2+^, Mg, BUN, creatinine, and HbA1c − Thyroid function tests − Liver function tests − Serum ferritin − Transferrin saturation − Disease-specific recommendations	− Plasma BNP and serum NT-proBNP for: diagnosis (I-A); severity (I-A); prognosis assessment (I-A); efficacy evaluation (IIa-B); screening (IIa-C) − Plasma A-type NP for: diagnosis (I-A); severity (IIa-B); prognosis assessment (IIa-B); efficacy evaluation (IIb-C); screening (IIb-C) − Myocardial troponins (T,I) and plasma noradrenaline for: severity (IIa-B); prognosis assessment (IIa-B) − Aldosterone and plasma renin activity for: severity (IIa-C); prognosis assessment (IIa-C) − Other neurohumoral factors for: severity (IIb-C); prognosis assessment (IIb-C)
Multimodality cardiovascular imaging	*Echocardiography*: − Evaluation of dimensions, LV and RV systolic (global and regional) and LV diastolic function at initial evaluation and follow-up (I-B) *CMR* − CMR at initial evaluation (I-B) − CMR during follow-up to monitor disease progression and aid risk stratification (IIa-C) − CMR in families in which a disease-causing variant has been identified for G+/P− family members (IIa-C) − CMR in P− family members without a genetic diagnosis (IIb-C) *CT/nuclear* − CCTA for patients who have inadequate echocardiographic imaging and contraindications to CMR (IIa-C) − CT-based imaging to exclude congenital or acquired CAD (IIa-C) − ^18^F-FDG-PET scanning if sarcoidosis is suspected (IIa-C)	*Echocardiography*: − PPCM: Echocardiography is commonly used to evaluate suspected PPCM, showing moderate to severe LV systolic dysfunction, LV dilatation, four-chamber enlargement, mitral and tricuspid regurgitation, biatrial enlargement, elevated pulmonary pressures, and RV enlargement − Chemotherapeutic-related CMP: Common methods for assessing cardiac function include echocardiography. Echocardiograms are preferred for regular monitoring as they avoid radiation and provide information on diastolic function haemodynamics, and pericardial disease, as well as valvular function. *CMR* CMR with T2 imaging for functional and viability assessment *CT* To assess extracardiac involvement in systemic diseases	*Echocardiography*: − To assess cardiac function, LV wall motion, valvular disease, RV function, and PH in suspected and diagnosed DCM with a change in clinical status (I-C) − To determine or modify treatment and for detection of cardiac thrombus (I-C) − For differential diagnosis (I-C) − As a follow-up procedure for clinically stable DCM (IIa-C) *CMR* − To assess cardiac anatomy and function (I-A) − LGE module to distinguish ischaemic from non-ischaemic scarring and identify underlying heart disease (IIa-B), and to predict arrhythmic events, mortality, and improvement in cardiac function (IIa-C) − T1 mapping indices to assess myocardial fibrosis (IIa-C) *CT/nuclear* − CT to differentiate ischaemic CMP via CCTA and to evaluate LV enlargement and thrombus. − 201 Tl and 99 mTc SPECT to assess myocardial viability; ECG-gated SPECT for LV volume and LVEF (IIa-B) − I-123-BMIPP and I-123-MIBG scintigraphy to predict prognosis (IIb-C)
Endomyocardial biopsy	When other clinical investigations suggest myocardial inflammation, infiltration, or storage that cannot be identified by other mean (IIa-C)	Can be useful in HF if it influences therapy, particularly when the diagnosis remains unconfirmed by other modalities	In patients with dilated HF where secondary CMP is suspected and remains undefined by other clinical examinations (IIa-C)
Genetic counselling and testing	*Genetic counselling* − Counselling for families with an inherited or suspected inherited CMP (I-B), with access to an expert MDT (I-B) − Pre- and post-test genetic counselling in all individuals (I-B) − Early in pregnancy if the family opts for pre-natal diagnostic testing (I-C) − Reproductive genetic testing discussion for all families with a genetic diagnosis (IIa-C) *Proband genetic testing* Where it enables CMP diagnosis, prognostication, therapeutic stratification, reproductive management, or cascade genetic testing (I-B) − Post-mortem genetic diagnosis if it aids in managing surviving relatives (I-C) − When diagnosis benefits the patient, considering psychological impact and preference (IIb-C) − Borderline phenotype not fulfilling diagnostic criteria after detailed assessment (IIb-C) *Family members* − Cascade genetic testing with pre- and post-test counselling in adult at-risk relatives (I-B) and in paediatric at-risk relatives (IIa-B) if established confident genetic diagnosis in a family individual. − Testing for a familial variant of unknown significance in parents and/or affected relatives (IIa-C) − Not recommended in a P– relative in the absence of a confident genetic diagnosis in the family (III-C)	*Genetic counselling and testing* − Genetic testing and genetic counselling in patients with familial or idiopathic CMP (LoE-B); *moderate level of consensus* − Genetic testing in familial DCM to confirm diagnosis, facilitate cascade screening, and help with family planning (LoE-A); *moderate level of consensus* *Family members* − Mutation-specific genetic testing for relatives after identifying a DCM-causative mutation (LoE-B); *strong level of consensus*	*Genetic counselling* For all patients with DCM regardless of family screening (I-B) − When receiving a genetic testing (I-B) − By professionals trained in genetics (IIa-C) *Genetic testing* − Detection of truncating variant in titin gene (IIa-C) − Detection of lamin A/C gene variant (I-C) especially in patients with DCM associated with conduction disturbance
Cardiac transplantation	Patients with advanced HF (NYHA Class III–IV) or intractable ventricular arrhythmia, without absolute contraindications (I-C)	− Autoimmune CMP: advanced HF without significant extracardiac of autoimmune disease (LoE-B) − Paediatric DCM: end-stage DCM HF treatment-resistant (LoE-B)	Severe HFrEF resistant to drug therapies and device treatments (IIa-C)
Left ventricular assist device	MCS for advanced HF patients suitable for heart transplantation despite optimal pharmacological and device treatment (IIa-B) or who are not eligible for heart transplantation or other surgical options, and without severe right ventricular dysfunction (IIa-B)	− PPCM: temporary or bridge, when medical therapy fails, and continuous inotropic therapy would be required (LoE B) − Autoimmune CMP: patients with advanced HF in the absence of significant extracardiac autoimmune disease (LoE B)	Treatment with implantable LVADs in patients with stage D HFrEF who were eligible for heart transplantation (IIa-C)
Atrial fibrillation/flutter management	*Anticoagulation*: OAC in all patients with DCM and AF or atrial flutter with a CHA_2_DS_2_-VASc score ≥2 in men or ≥3 in women (I-B) or a CHA_2_DS_2_-VASc score of 1 in men or 2 in women (IIa-B)^c^ *Control of symptoms and HF*: − AF catheter ablation for RC after one failed or intolerant Class I or III AADs (I-B) or as first-line RC therapy (IIa-C) in patients with paroxysmal or persistent AF − AF catheter ablation to reverse LV dysfunction when tachycardia-induced component is highly probable (I-B) − Maintenance of SR rather than RC at an early stage in AF patients without major risk factors for recurrence (IIa-C) − AF catheter ablation in patients with AF, HF, and/or reduced LVEF (IIa-B) *Risk factor management*: − Modification of unhealthy lifestyle and targeted therapy of intercurrent conditions (I-B)	NR	*HF with AF*: Rate control (I-C) with: − β-Blockers (I-A) − Digitalis (IIa-B) − Amiodarone in case of insufficient with β-blockers or digitalis (IIb-C) − Ablation of the AV node if pacemaker therapy is allowed (IIb-C) − Non-DHP CCBs (III-C) Rhythm control (I-C) − Amiodarone (IIb-B) − Catheter ablation (IIa-B) − Sodium-channel blockers are contraindicated (III-A)
SCD risk stratification and ICD implantation	*General recommendations*: − In patients who have an expectation of good quality survival >1 year (I-C) − Evidence-based shared decision-making that considers preferences and comprehension of benefits and harms (I-C) − Counselling on risks of inappropriate shocks, implant complications, and device impacts (I-C) − Not recommended in patients with incessant VA, until it is controlled (III-C) *Secondary prevention*: − In patients who have survived a CA due to VT or VF, or who have spontaneous sustained VA causing syncope or haemodynamic compromise in the absence of reversible causes (I-B) − In patients presenting with haemodynamically tolerated VT, in the absence of reversible causes (IIa-C)	− PPCM: ICD, CRT, or both should be considered for patients with PPCM whose ventricular function does not normalize after pregnancy according to current guidelines (LoE B); *strong level of consensus* − Paediatric DCM: ICDs can be useful in high-risk patients with DCM to prevent sudden death (LoE C); *moderate level of consensus*	− Patients with VF without reversible factors (I-A) − SVT without reversible factors with ≥1 of the following (I-C): • Syncope in association with VT • BP ≤80 mmHg, symptoms of cerebral ischaemia or chest pain in association with VT • Polymorphic VT • Haemodynamically stable monomorphic VT with ineffective or contraindicated pharmacotherapy, unassessable drug efficacy, or ineffective/infeasible catheter ablation. − No longer inducible SVT after catheter ablation (IIa-B) − SVT with effective therapy established by follow-up and drug efficacy evaluation (IIa-B) − Patients prone to VT or VF due to an acute reversible disorder (e.g. myocardial ischaemia, electrolyte imbalance, and drugs) and at high risk of re-exposure despite treatment (IIa-C)
	*Primary prevention:* − Comprehensive SCD risk stratification for patients with prior CA or VA, initially and every 1–2 years or with clinical status changes (I-C) − Use of validated SCD algorithms/score to the shared decision-making (IIa-B) − If a patient needs a pacemaker, conduct comprehensive SCD risk stratification for potential ICD implantation (IIa-C) − In patients with DCM, symptomatic HF and LVEF ≤35% despite >3 months of OMT (IIa-A) − Patient's genotype consideration in the estimation of SCD risk (IIa-B) − DCM with a genotype associated with high SCD risk and LVEF >35% + additional risk factors: LMNA, FLNC-truncating variants, TMEM43, PLN, DSP, RBM20 (IIa-C) − DCM with a genotype associated with high SCD risk and LVEF >35% without additional risk factors (IIb-C) − DCM without a genotype associated with high SCD risk and LVEF >35% in the presence of additional risk factors (IIb-C) *Choice of ICD*: − Evaluate the needs of CRT (I-A) − S-ICD as an alternative to TV-ICD when need for anti-bradycardia pacing, CRT, or ATP is not anticipated (IIa-B) − WCD for adults with a secondary prevention indication who are temporarily not candidates for ICD implantation (IIa-C)		− Patients who meet all the following (I-A): • After ≥3 months of optimal medical therapy • Symptomatic HF (NYHA II—III) • LVEF ≤35% • Non-sustained VT or unexplained syncope − Patients who meet all the following conditions (IIa-B): • After ≥3 months of optimal medical therapy • Symptomatic HF (NYHA II and III) • LVEF ≤35% − Patients with unexplained syncope and LVEF ≤35% (IIa-C) − An indication of WCD for patients who meet all the following conditions (IIa-C): • Received new medical therapy for HF (within 90 days) • An increased risk of SCD • Having possibility to recover LV systolic function − Contraindicated in patients who meet ≥1 of the following (III-C): • Unable to express consent or cooperate with treatment due to mental disorders or other reasons • Physical restriction for chronic diseases • Life expectancy <12 months • VT/VF with a known acute reversible disorder • Frequent VT/VF, which cannot be controlled with antiarrhythmic drugs and/or catheter ablation • Severe drug-resistant congestive HF and NYHA IV symptoms who are not indicated for heart transplantation, CRT, or LVA
Multidisciplinary teaming	− Access to MDTs for all patients and their relatives (I-C) − Transition of care from paediatric to adult services (I-C)	Based on a specific type of DCM, e.g.: − Anthracycline-induced CMP: oncologists, transplant team − PPCM: high-risk obstetricians, intensivists, neonatologists − Genetic CMPs: genetic counsellors, DNA storage experts, perinatologist	NR
Surveillance program	− Multiparametric approach including ECG and echocardiography for all clinically stable patients every 1–2 years (I-C) − ECG and MMI in patients with change in symptoms (I-C)	*Strong level of Consensus*: − PPCM: Annual LVEF assessments, for several years after recovery (LoE-B). Pregnant women should be referred to a specialized centre for multidisciplinary care and close monitoring around delivery (LoE-C) − CMPs related to chemotherapeutic agents: measure LVEF at baseline, after treatment, regularly during treatment, or sooner if HF symptoms develop (LoE-B). If cardiac function deteriorates, weigh the therapy's benefits against the risk of irreversible damage (LoE-C) *Uncertain level of consensus*: − CMPs related to chemotherapeutics agents: serial measurements of cardiac biomarkers for monitoring cardiotoxicity (LoE-C)	NR
Relatives screening and follow-up	− Following cascade genetic testing, multiparametric approach including ECG and cardiac imaging and long-term follow-up in first-degree relatives who have the same disease-causing variant as the proband (I-B) − Following cascade genetic testing, follow-up dischargement for P-first-degree relatives who do not have the same disease-causing variant as the proband (I-C) − Initial evaluation (I-C) and regular long-term (IIa-C) with multiparametric approach including ECG and cardiac imaging in first-degree relatives when no P/LP variant is identified in the proband, or genetic testing is not performed − During cascade screening, clinical evaluation of close relatives when a first-degree relative has died (IIa-C)	− Periodic serial echocardiographic screening with assessment of LV function and size in first-degree relatives of patients with familial CMP (LoE C); *strong level of consensus* − Paediatric DCM: In first-degree relatives of paediatric-DCM, ECG and echocardiographic screening for cardiomyopathy can be beneficial (LoE-C); *moderate level of consensus* − Periodic serial echocardiography screening with assessment of LV function and size for first-degree relatives of patients with idiopathic CMP (LoE-C*); uncertain level of consensus*	NR
Psychological support in patients and family members	− Psychological support to all individuals experiencing SCD of a family member (I-B) − Psychological support to all individuals who receive an ICD (I-B) − Psychological support to all individuals experiencing new diagnosis, exercise restrictions, symptomatic disease, and genetic testing (IIa-C)	NR	− Discuss diagnosis naming with healthcare professionals and conduct regular mental screenings, considering lifelong disease management and fear of sudden death − These patients should also be given assistance acquiring counselling as needed
Exercise recommendations	− Regular low-to moderate-intensity exercise in all able individuals (I-C) − Individualized risk assessment for exercise prescription (I-C) − Moderate- and high-intensity exercise in G+/P− individuals, except for LMNA+ and TMEM43+ (IIa-C) − High-intensity exercise and competitive sports in a select group of asymptomatic and optimally treated individuals with LVEF ≥50% in absence of exercise-induced complex arrhythmias (IIb-C) − Moderate-intensity exercise in asymptomatic and optimally treated individuals with an LVEF of 40–49% in absence of exercise-induced complex arrhythmias (IIb-C) − High-intensity exercise not recommended in symptomatic individuals, LVEF ≤40%, exercise-induced arrhythmias or LMNA+ or TMEM43+ (III-C)	*Strong level of consensus*: − Exercise training is recommended for patients with HF who are able, including those who are obese (LoE-B).	− Combination with drug therapy to relieve symptoms and improve exercise capacity (I-A)^d^ − Exercise to improve QOL, reduce cardiac accidents, and improve life expectancy (IIa-B)^d^ − For patients with advanced deconditioning and patients with reduced physical function, resistance training to improve ADL and QOL by increasing muscle strength and endurance (IIa-C)^d^ − Exercise training is recommended for patients with stable chronic HF, when excluded contraindicated conditions. It is desirable to perform exercise test and to prescribe exercise therapy based on the results^d^
Reproductive issues	− Pre-pregnancy risk assessment and counselling in all women using the mWHO classification of maternal risk (I-C) − Counselling on safe and effective contraception in all women of fertile age and their partners (I-C) − Counselling on the risk of disease inheritance for all men and women before conception (I-C) − Vaginal delivery in most women with severe HF (LVEF <30% or NYHA III and IV), or severe outflow tract obstructions, or women in labour on oral anticoagulants (I-C) − Careful medication review for safety and tolerability before pregnancy (I-C) − Anticoagulation with LMWH or VKAs according to the trimester of pregnancy in patients with AF (I-C) − β-Blocker continuation with close follow-up of foetal growth if benefits outweigh risks (IIa-C) − Genetic counselling and testing in patients with PPCM (IIa-C)	*Strong level of consensus*: − PPCM: Patients whose ventricular function does not normalize after pregnancy should be counselled against a subsequent pregnancy, because it carries a significant risk of morbidity and mortality (LoE-B) − Prompt delivery is recommended for pregnant women with PPCM whose condition is unstable or who have maternal extremis (LoE-B) *To avoid*: − PPCM: In women with a history of PPCM and persistent LV dysfunction, subsequent pregnancy is contraindicated (LoE-C)	− HF (NYHA III and IV, LVEF <35–40%) requires strict monitoring during pregnancy or a strong recommendation to avoid pregnancy − Intensive specialist cardiac and obstetric monitoring are needed throughout pregnancy, childbirth, and the puerperium − If LVEF <20%, the pregnancy should be terminated − In high-risk patients, frequent echocardiography is needed − ACEi and ARBs are contraindicated. Aldosterone antagonists and β-blockers can be used during pregnancy (careful monitoring of foetus and newborn) − Diuretics, carperitide, and catecholamine can be used for AHF during pregnancy − Delivery by caesarean section should be considered in patients with poorly controlled HF − In patients with deteriorated cardiac function, vaginal delivery with epidural anaesthesia is recommended to reduce the cardiac load
Non-cardiac surgery	− Peri-operative ECG monitoring for all patients undergoing surgery (I-C) − When scheduled for intermediate- or high-risk NCS, re-evaluation of LV function with echocardiography and NT-proBNP/BNP levels assessment (I-B) − Performing ECG and TTE before NCS, regardless of symptoms, in all patients <65 years and a first-degree relative with CMP (I-C)	NR	The assessment of exercise tolerance deemed useful for stratification of cardiovascular risk during surgery
Management of cardiovascular risk factors	Identification and management of risk factors and concomitant diseases recommended as an integral part of the management (I-C)	− Optimization of hypertension in patients with autoimmune-mediated HF (LoE-C); *strong level of consensus* − Weight loss for managing comorbidities in obese patients with HF (LoE-C); *uncertain level of consensus* − Weight reduction is of uncertain benefit to reduce morbidity or mortality in HF (LoE-B); *uncertain level of consensus:*	− Smoking cessation (I-C)^e^ − Low-salt diet (IIa-C)^e^ − Restriction of alcohol (IIa-C)^e^ − Patients should be instructed to follow a balanced diet to maintain an appropriate body mass index as well as good hydration^e^

Class of recommendations: I, conditions for which there is evidence for and/or general agreement that the procedure or treatment is beneficial, useful, and effective; II, conditions for which there is conflicting evidence and/or a divergence of opinion about the usefulness/efficacy of a procedure or treatment; IIa, weight of evidence/opinion is in favour of usefulness/efficacy; IIb, usefulness/efficacy is less well established by evidence/opinion; III, conditions for which there is evidence and/or general agreement that the procedure/treatment is not useful/effective and in some cases may be harmful.

Level of evidence: A = Data derived from multiple randomized clinical trials or meta-analysis. B = Data derived from a single randomized trial or non-randomized studies. C = Only consensus opinion of experts, case studies, or standard of care.

^18^F-FDG-PET, ^18^F-fluorodeoxyglucose positron emission tomography; AAD, antiarrhythmic drug; ACE, angiotensin-converting enzyme; AF, atrial fibrillation; AHA, American Heart Association; AHF, acute heart failure; A-type ANP, atrial (A-type) natriuretic peptide (ANP); BP, blood pressure; BUN, blood urea nitrogen; CA, cardiac arrest; CAD, coronary artery disease; CCTA, coronary computed tomography angiography; CMR, cardiovascular magnetic resonance; CHA2DS2-VASc, Congestive heart failure or left ventricular dysfunction, Hypertension, Age ≥75 (doubled), Diabetes, Stroke (doubled)-vascular disease, Age 65–74, Sex category (female) (score); CHD, congenital heart disease; CK, creatinine kinase; CMP, cardiomyopathy; CT, computed tomography; DCM, dilated cardiomyopathy; EACVI, European Association of Cardiovascular Imaging; EI, editorial independence declared; ESC, European Society of Cardiology; FFAs, free fatty acids; G, genotype; HbA1c, haemoglobin A1c; HF, heart failure; HFrEF, heart failure with reduced ejection fraction; ICD, implantable cardioverter defibrillator; JCS, Japanese Circulation Society; LMWH, low-molecular-weight heparin; LoE, level of evidence; LVAD, left ventricular assist device; LVEF, left ventricular ejection fraction; MCS, mechanical circulatory support; MDT, multidisciplinary team; MMI, multimodality imaging, mWHO, modified World Health Organization; NCS, non-cardiac surgery; non-DHP CCBs, non-dihydropyridine calcium antagonists; NR, not reported; NT-proBNP, N-terminal pro-brain natriuretic peptide; NYHA, New York Heart Association; OAC, oral anticoagulation; OMT, optimal medical therapy; P, phenotype; PH, pulmonary hypertension; P/LP, pathogenic/likely pathogenic; PPCM, peripartum cardiomyopathy; RC, rhythm control; SCD, sudden cardiac death; SCI, statement about conflicts of interest of group members present; S-ICD, subcutaneous implantable cardioverter defibrillator; SPECT, single photon emission computed tomography; SR, sinus rhythm; TIA, transient ischaemic attack; TTE, transthoracic echocardiogram; TV-ICD, transvenous implantable cardioverter defibrillator; VA, ventricular arrhythmias; VF, ventricular fibrillation; VKA, vitamin K antagonist; VT, ventricular tachycardia; WCD, wearable cardioverter defibrillator.

^a^Relationship with industry is reported by any group member.

^b^A group member is reported recused when a relevant area is under discussion.

^c^According to the 2024 ESC guidelines for the management of AF, the CHA2DS2-VASc score no longer includes the sex category and is now referred to as the CHA2DS2-VA score. Accordingly, OACs are recommended in patients with clinical atrial fibrillation/flutter and a CHA_2_DS_2_-VA score of ≥2 (Class I, level C) and should be considered in those with clinical atrial fibrillation/flutter and a CHA_2_DS_2_-VA score of 1 (Class IIa, level C), following a patient-centred and shared care approach—https://doi.org/10.1093/eurheartj/ehae176.

^d^Since there is no distinction in exercise training protocols for HFrEF patients in daily clinical practice, the following information applies to both HFrEF and DCM.

^e^Recommendation based on HF guidelines.

## Areas of agreement

### Definition

All the included documents provided a consistent definition of DCM as a spectrum of myocardial disorders that are characterized by ventricular dilatation and impaired myocardial systolic function unexplained solely by abnormal loading conditions.^1,10,11^ However, only the European guidelines specify that right ventricular dilatation and dysfunction may be present but are not required for the diagnosis.^1^

### Diagnostic workup

There was consensus that a multiparametric approach is essential for evaluating suspected or established cardiomyopathies. This approach includes clinical evaluation, a detailed history, ECG, laboratory tests, and multimodality cardiovascular imaging. The guidelines also agree on the importance of conducting a thorough pedigree analysis and obtaining a three-to-four-generation family history, as these are crucial for an accurate diagnosis. All guidelines and recommendations endorse the use of B-type natriuretic peptide (BNP) and troponin assessments as primary diagnostic tools, not only for initial diagnosis but also for evaluating severity, prognosis, and response to treatment. Regarding invasive assessments, all guidelines recommend considering endomyocardial biopsy when other clinical investigations suggest an underlying cause that has not been identified through other examinations, and where targeted therapy may be beneficial.^1,10,11^

### Multimodality cardiovascular imaging

There was agreement across all three documents on the critical role of multimodality imaging in the diagnostic workup for DCM. Each guideline recommends transthoracic echocardiography (TTE) as the first-line imaging test for assessing global and regional LV anatomy, function, and haemodynamics, as well as for evaluating valvular heart disease, right heart function, and associated features during both the initial evaluation and follow-up. However, the guidelines do not provide specific thresholds for the degree of LV dilatation or dysfunction necessary to diagnose DCM. Additionally, cardiac magnetic resonance (CMR) is universally recommended for its ability to assess cardiac function and tissue characterization during the initial evaluation, as well as for identifying specific cardiomyopathy phenotypes and distinguishing them from phenocopies. All documents underscore the importance of using complementary imaging modalities to achieve a comprehensive evaluation of the disease.

### Genetics testing and counselling

Notably, although beyond the methodological scope of this project, it is key to highlight that some documents emphasize the importance of tailored approaches in the management and prognostic stratification of cardiomyopathies, based on genetic factors. For example, Hershberger *et al*.^2^ stress the importance of a systematic genetic approach to cardiomyopathy evaluation, beginning with a comprehensive family history to guide test selection and interpretation. This process extends to referral to expert centres capable of providing thorough pre- and post-test genetic counselling.^12^ As for our analysis, all societies concur that genetic testing and counselling have become standard practices in managing individuals with DCM and their families. The ESC guidelines provide a Class I recommendation for genetic testing, emphasizing its role in diagnosing DCM, assessing prognosis, guiding therapy, managing reproductive decisions, and conducting cascade genetic testing,^1^ while the AHA scientific statement delivers moderate consensus on genetic testing.^10^ Additionally, the ESC guidelines recommend genetic testing post-humously if it aids in the management of surviving relatives.^1^ Both the European and Japanese guidelines agree that first-line genetic testing in a proband should target genes strongly associated with the presenting phenotype.^1,11^ The Japanese guidelines specifically issue a Class I recommendation for detecting lamin A/C (LMNA) gene variants, particularly in DCM patients with conduction disturbances, such as atrioventricular block, and a Class IIa recommendation for identifying truncating variants in the titin (TTN) gene.^11^ For family members, both the AHA and the ESC strongly recommend cascade family screening using genetics for at-risk relatives once a pathogenic or likely pathogenic (P/LP) variant is established in a family member with cardiomyopathy.^1,10^ The European guidelines uniquely specify that genetic testing is not recommended for phenotype-negative relatives without a confirmed family genetic diagnosis.^1^ There is unanimous agreement across the recommendations that careful genetic counselling is mandatory for individuals with inherited or suspected inherited DCM.^1,10,11^ The European guidelines stand out by delivering a strong Class I recommendation for pre- and post-test counselling, as well as early genetic counselling for women considering pre-natal diagnostic testing. They also provide a Class IIa recommendation for discussing reproductive genetic options in families with a confirmed genetic diagnosis. Furthermore, the European guidelines recommended involving an appropriately trained healthcare professional for genetic counselling as a Class IB recommendation,^1^ while the Japanese guidelines offer a weaker Class IIa C recommendation.^11^ The AHA scientific statement, however, does not specify the professional roles that should be included in the genetic counselling team.^10^

### Advanced heart failure management

All the included documents recommend cardiac transplantation for patients with advanced HF [New York Heart Association (NYHA) Class III and IV] who are refractory to medical and device therapies and do not have absolute contraindications. The guidelines also agree that mechanical circulatory support (MCS) should be considered for patients eligible for heart transplantation to improve symptoms and reduce the risk of hospitalization and death while awaiting a transplant.^1,10,11^ However, only the European guideline provided a Class IIa recommendation for MCS as a long-term treatment option in patients who are not eligible for heart transplantation or other surgical interventions, provided they do not have severe right ventricular dysfunction.^1^

## Areas of disagreement

### Multidisciplinary team approach

There were inconsistencies in the recommendations regarding the collaboration of different specialties in managing these complex patients. Only the ESC guidelines provide a Class I recommendation for access to a multidisciplinary team (MDT). These teams should include cardiomyopathy specialists, patient support services, and other relevant specialties to comprehensively manage both patients and their relatives, including during the transition from paediatric to adult care.^1^ The AHA offers only general statements on this matter,^10^ while the JCS guidelines do not address it at all.^11^ Further, only the ESC guidelines recommend that genetic testing should involve an MDT with expertise in genetic testing methodology, variant interpretation, and clinical application, ideally within a specialized cardiomyopathy service or equivalent network.

### Imaging and laboratory biomarkers

Although the documents agree on the use of TTE and CMR, there are discrepancies regarding the role of computed tomography (CT) and nuclear imaging. The ESC guidelines recommend coronary computed tomography angiography (CCTA) for patients with poor echocardiographic acoustic windows or contraindications to CMR, and for ruling out CAD.^1^ In contrast, the AHA suggests a broader application of CT, including the assessment of extracardiac involvement. The JCS guidelines are more specific, advocating for the use of CCTA to differentiate ischaemic cardiomyopathy and to evaluate intracardiac thrombus and LV dilatation.^11^ Regarding nuclear imaging, the ESC recommends ^18^F-fluorodeoxyglucose positron emission tomography (^18^F-FDG-PET) scanning in cases of suspected sarcoidosis,^1^ while the AHA statement does not mention nuclear imaging applications.^10^ The JCS guidelines, on the other hand, incorporate a wide range of nuclear imaging techniques, including 201Tl and 99mTc single photon emission computed tomography (SPECT) for myocardial viability, ECG-gated SPECT for LV function, and 15-(4-^123^I-iodophenyl)-3(*R,S*)-methylpentadecanoic acid (I-123-BMIPP) and ^123^I-metaiodobenzylguanidine (I-123-MIBG) scintigraphy for prognosis prediction, demonstrating a broader range of clinical indications in DCM patients.^11^ In terms of laboratory tests, while there is consensus on the assessment of BNP and cardiac troponin, discrepancies remain regarding other ancillary tests. The ESC guidelines strongly recommend, with a Class I recommendation, routine (first-level) laboratory tests for all patients, including assessments for aetiology, disease severity, and extracardiac manifestations. They also suggest, with a Class IIa recommendation, that additional (second-level) tests should be conducted in selected patients to aid in detection of metabolic and syndromic causes, following specialist evaluation, and to identify specific aetiologies.^1^ In contrast, the AHA statement emphasizes these laboratory tests as part of the routine workup during the initial evaluation of DCM patients.^10^ Meanwhile, the JCS guidelines place additional emphasis on biomarkers such as plasma atrial natriuretic peptide (ANP), aldosterone, plasma renin activity, and other neurohumoral factors for diagnosis and prognosis assessment.^11^

### Genetic screening and cascade testing

There is variation in guidance on genetic screening and cascade testing for DCM. The ESC guidelines strongly recommend testing other family members for the identified causative variant once a confident genetic diagnosis has been established in one affected individual, irrespective of age. They provide a Class I recommendation for clinical evaluation and long-term follow-up of first-degree relatives who carry the same variant as the proband. In contrast, phenotype-negative first-degree relatives who do not carry the same variant are advised to be discharged from further follow-up.^1^ Additionally, the ESC guidelines recommend clinical evaluation of close relatives of the deceased (i.e. second-degree relatives of the proband) in cases where a first-degree relative has died. No other guidelines provide information on this matter.^1^

### Risk stratification

While risk stratification and the subsequent indications for implantable cardioverter defibrillator (ICD) implantation are largely informed by HF guidelines, the reviewed documents display notable inconsistencies in the presentation of recommendations and identification of low-, intermediate-, and high-risk patient subgroups. Notably, the ESC guidelines emphasize general recommendations, such as assessing patient life expectancy (>1 year), engaging in shared decision-making that considers patient preferences, carefully balancing the potential benefits against the risks, and thoroughly informing patients about potential complications^1^—areas not equally highlighted in other guidelines. With regards to peripartum cardiomyopathy (PPCM), the AHA guidelines explicitly recommend ICD implantation for patients whose LV function fails to recover post-delivery. Meanwhile, the ESC guidelines provide a less definitive stance, suggesting higher thresholds for ICD implantation in PPCM patients to account for the potential for spontaneous post-delivery recovery.^1^ While there is consensus across the three documents regarding ICD implantation in patients who have survived sudden cardiac arrest due to malignant ventricular arrhythmias—provided these arrhythmias are not attributable to reversible causes—the ESC, JCS, and AHA offer contrasting recommendations on wearable cardioverter defibrillators (WCDs). The ESC exhibits caution, supporting WCDs mainly for secondary prevention in patients who are temporarily ineligible for ICDs, noting a lack of data for primary prevention in conditions other than early post-myocardial infarction, like myocarditis and PPCM.^1^ In contrast, the JCS views WCDs as an effective bridge therapy for primary prevention in patients who have yet to complete the recommended 90 days of HF therapy before an ICD implantation is considered.^11^ Meanwhile, the AHA recommends WCDs for primary prevention in high-risk PPCM patients with left ventricular ejection fraction (LVEF) <35% who are still assessing their response to medical therapy and do not yet have a clear indication for an ICD.^10^ These differing perspectives underline varied clinical approaches and considerations regarding the timing and appropriateness of WCD deployment in preventing sudden cardiac death (SCD). A primary area of disagreement concerns the role of genetics in arrhythmic risk stratification, which is extensively highlighted in the ESC guidelines. The advent of risk stratification tools, such as LMNA^13^ and phospholamban (PLN)^14^ risk scores, is encouraged to support shared decision-making processes and underscore the importance of genetic factors in the determination of cardiac pathology. For instance, the ESC guidelines recommend ICD implantation with a Class IIa indication for genotype-positive probands with LMNA, FLNC-truncating variants, PLN, DSP, or transmembrane protein 43 (TMEM43), even when LVEF is <50% but >35%—a long-established threshold for ICD candidacy.^1^

### Non-cardiac surgery management

Only the ESC guidelines provide specific recommendations for the management of non-cardiac surgery (NCS). They offer a Class I recommendation for peri-operative ECG monitoring in patients scheduled for surgery, re-evaluation of LV function with TTE, and assessment of BNP/amino-terminal pro-BNP (NT-proBNP) levels for those undergoing intermediate- to high-risk NCS. Additionally, they advise performing ECG and TTE prior to NCS in all patients younger than 65 years and in first-degree relatives with cardiomyopathy, irrespective of symptoms.^1^ In contrast, the JCS guidelines offer only general statements, recommending the use of exercise tolerance assessments for cardiovascular risk stratification in the peri-operative setting.^11^

## Discussion

**Figure 2:**
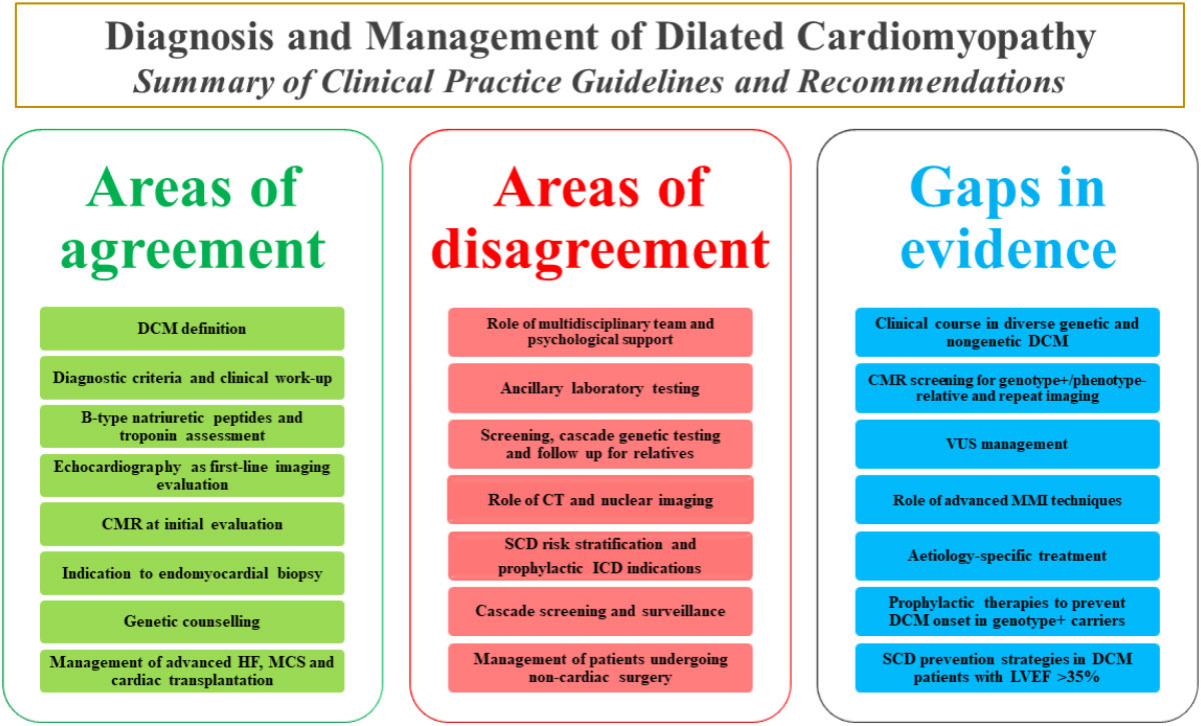
Summary of clinical practice guidelines and recommendations on dilated cardiomyopathy diagnosis and management. CMR, cardiovascular magnetic resonance; CT, computed tomography; DCM, dilated cardiomyopathy; G, genotype; HF, heart failure; ICD, implantable cardioverter defibrillator; LVEF, left ventricular ejection fraction; MCS, mechanical circulatory support; MMI, multimodality imaging; P, phenotype; SCD, sudden cardiac death; VUS, variant of unknown significance.

We identified two clinical practice guidelines and a scientific statement, which were all rigorously developed, on the diagnosis and management of patients with DCM. A large proportion of the recommendations were noted to be based on expert consensus over evidence (level of evidence C). There is consensus on the definition of DCM and the role of natriuretic peptides together with troponin assessment for laboratory workup. TTE and CMR share common recommendations at first-line imaging modalities for evaluation in patients with suspected cardiomyopathy. All documents agree on the role of genetic counselling, testing, and management of advanced HF, MCS, and cardiac transplantation. Variations in the recommendations are present in the role of MDTs, psychological support, cascade genetic testing, SCD risk stratification, and prophylactic ICD recommendations. *Figure 2* provides a summary of the areas of agreement, disagreement, and gaps in evidence that may shed a light on future research and guideline development. Despite advances, several key areas in DCM management remain inadequately addressed. There is a need for a better understanding of the clinical trajectory of diverse genetic and non-genetic forms of DCM, particularly regarding clinical descriptors, disease penetrance, and long-term outcomes. The role of CMR screening for genotype-positive/phenotype-negative relatives and the appropriate timing for repeat imaging are still unclear, as is the management of variants of unknown significance (VUS). Furthermore, the potential of advanced multimodal imaging techniques in refining risk stratification and guiding therapy warrants further exploration. Aetiology-specific treatments remain a critical gap, with most current therapies not targeting the root cause of the disease. There is also a pressing need for effective prophylactic strategies to prevent DCM onset in genotype-positive carriers and to develop more precise SCD prevention strategies for DCM patients with LVEF between 35% and 50%. In the following sections, we will explore these areas of concern in greater depth, specifically focusing on defining the clinical course of DCM, strategies for managing VUS, and the development of aetiology-oriented therapies.

### Defining dilated cardiomyopathy clinical course

Advances in cardiogenetics, alongside the ClinGen classification for a diagnostic approach to cardiomyopathies and the growing emphasis on genotype–phenotype correlations to enhance arrhythmic risk stratification in DCM patients, have contributed to a more precise understanding of the disease's underlying pathophysiological mechanisms. However, significant gaps in evidence regarding the clinical course of DCM remain.^15–18^ Within the ESC EORP Cardiomyopathy and Myocarditis registry, a prospective analysis was conducted to elucidate the phenotypic differences, clinical management strategies, and outcomes between patients with familial and sporadic DCM. Despite significant variations in baseline characteristics and disparities in access to genetic counselling, the short-term prognosis between these groups revealed no statistically significant differences.^19^ A subsequent analysis, drawing on updated data from the same European registry, has further refined our understanding of DCM's clinical trajectory, with particular attention to disease onset, familial patterns, and geographical variations in diagnosis. Notably, relatives who were diagnosed at a younger age compared with probands yet showed a similar timeline for symptom onset and indications for ICD implantation. Additionally, patients from Southern Europe were observed to have a higher incidence of familial transmission, were more likely to be diagnosed through family screening, and presented more often with rare underlying conditions.^20^ The advent of advanced multimodal imaging techniques has considerably improved the precision of prognostic stratification, particularly when combined with guideline-directed medical therapy (GDMT) for HF, which has proved effective in inducing LV reverse remodelling. In patients with idiopathic DCM, the absence of late gadolinium enhancement, lower baseline T2 mapping, and extracellular volume values have emerged as independent predictors of LV reverse remodelling, paralleled by reductions in native myocardial T1, matrix, and cell volume.^21^ Despite significant advancements, DCM remains a leading cause of mortality and morbidity, with a diverse and often unpredictable phenotypic spectrum. Moving forward, ‘imagenetics’ or ‘imagenomics’, that is the combination of advanced multimodality imaging and cardiogenetics, may be pivotal for the precise selection of patients, facilitating early detection and the deployment of targeted therapies to effectively halt/slow disease progression.^8^ Ongoing randomized trials such as the CMR-GUIDE,^22^ ReCONSIDER,^23^ or BRITISH^24^ are expected to provide important answers on the optimal approach for primary SCD prevention in DCM. Additionally, the potential of gene editing offers a promising avenue for future interventions that, merged with gene-first screening strategies,^25^ may significantly enhance the precision of tailored management approaches.

### Variants of unknown significance management

The role of genetics in the assessment of DCM is becoming increasingly important, particularly when combined with tissue characterization through multimodal imaging. This integration aids in providing a comprehensive evaluation of the disease, guiding highly personalized treatments, and identifying potential aetiological variants in close relatives. Currently, 12 genes are strongly associated with DCM, while 7 have a moderate association.^26^ The challenge in DCM genetic characterization lies in the variable penetrance of these genetic variants and their concomitant variable expressivity, where environmental factors—such as age, sex, comorbidities, and external triggers—play a crucial role. While the use of expanded gene panels has improved the ability to diagnose DCM, it has also led to an increase in the identification of VUS, which complicates interpretation and clinical management.^1^ Variants of unknown significance are often ‘private’ to the family being investigated, adding to the uncertainty about the variant.^27^ To mitigate this issue, *in silico* prediction tools, co-segregation analysis, and high-throughput *in vitro* cellular functional validation have contributed to the accurate interpretation of VUS. Today, there are also specialized web-based models that can assist in the differential diagnosis of gene variant assessment through amino acid-level signal-to-noise analysis.^28^ In a cohort of 902 DCM patients from the Maastricht Cardiomyopathy Registry, various genetic testing panels were employed to reclassify identified variants into more robust gene associations with the patient's cardiomyopathy phenotype. The results demonstrated that stringent gene selection for DCM genetic testing reduced the incidence of VUS while still effectively identifying P/LP variants.^29^ These findings suggest that focusing on a smaller, more robust set of genes in diagnostic panels may yield clearer and more clinically relevant outcomes for managing DCM. Familial risk of DCM among relatives of affected individuals depends on the degree of relationship, being strongest in full‐siblings but still significant in second‐degree and third‐degree relatives. This supports the idea that genetic components of DCM also exist in those cases where genetic testing fails to identify a major causative gene, indicating that more complex genetic backgrounds (e.g. mixed Mendelian or polygenic aetiology, unidentified environmental factors) may contribute to the susceptibility to cardiomyopathy.^30^ The role of epigenomics in this context has been only marginally explored. Structural modifications of DNA, such as methylation, have been associated with altered expression in genes involved in energy metabolism, impacting cardiomyocyte function.^31^ Currently, there is insufficient evidence regarding the integrated management of genetic testing for relatives in cases of VUS. However, an integrated omics approach, leveraging cutting-edge testing technologies, could enhance the understanding of disease pathogenesis and potentially guide tailored therapeutic strategies.

### Aetiology-oriented and disease-modifying therapies

All documents provide recommendations on appropriate stage classification, GDMT, and arrhythmic risk stratification, aiming to assess the need for ICD implantation to prevent SCD.^1,10,11^ However, the available treatment options cannot be considered aetiology-specific or disease-modifying, as they do not directly target the underlying cause of the disease. Recent insights have highlighted the limitations of using a strict LVEF cut-off of <35% as the primary criterion for ICD implantation. The ESC guidelines are progressively shifting away from this approach, acknowledging that LVEF alone may not adequately capture an individual's risk of SCD. The findings from the DANISH trial reinforce this perspective, showing that routine prophylactic ICD implantation did not improve survival in patients with non-ischaemic DCM and an LVEF <35%.^32^ Consequently, this LVEF-based strategy can lead to both overtreatment—where low-risk patients with an LVEF <35% receive unnecessary ICDs—and undertreatment—where patients with an LVEF ≥35% are denied ICDs despite having significant additional risk factors that increase their SCD risk.^33^ A more individualized risk stratification, incorporating genetic markers, advanced imaging techniques, and clinical history, is essential for accurately identifying those who would truly benefit from ICD implantation, thereby optimizing patient outcomes and avoiding unnecessary procedures. In line with this perspective, the ESC guidelines advocate for a personalized approach that considers high-risk variants more strongly associated with malignant arrhythmias (e.g. LMNA, PLN, FLNC-truncating variants, TMEM43, DSP, and RBM20) and other risk factors, such as the presence and extent of myocardial scarring determined by late gadolinium enhancement on CMR imaging. Additional risk factors, such as syncope or the presence of non-sustained ventricular tachycardias (NSVT), may also help guide ICD implantation.^1^ While the development and widespread application of gene-editing therapies are still awaited, trials are currently underway on two fronts. The EARLY-GENE Trial (NCT05321875) is investigating the potential of preventive GDMT to alter the clinical trajectory of the disease, even in the absence of overt systolic dysfunction or HF with reduced ejection fraction. Concurrently, a phase 2a trial (NCT04572893) is evaluating the safety and preliminary efficacy of danicamtiv, a selective allosteric activator of cardiac myosin, which may have a role in reversing or preventing LV systolic dysfunction in patients with primary DCM due to myosin heavy chain beta 7 (MYH7) or TTN variants. At present, the foremost unmet need in the therapeutic management of DCM is to move beyond symptomatic treatment and SCD prevention, and instead focus on directly targeting the underlying genetic and molecular mechanisms driving the disease. Various gene therapies are currently in development, including those aimed at myosin-binding protein C3 (MYBPC3)-related cardiomyopathy (NCT05836259) and the use of antisense oligonucleotides for PLN-R14del cardiomyopathy.^34^

## Sustainable strategies

In the management of DCM, aligning diagnostic and treatment strategies with both cost-effectiveness and environmental sustainability is increasingly important. However, it is noteworthy that none of the existing documents explicitly addresses these aspects in the so-called *cardiomyopathy pathway*. This oversight highlights a significant gap in current guidelines and practices. The precision offered by advanced genetic testing, for instance, allows for a more targeted approach to diagnosing and managing DCM, including family cascade screening and relatives’ follow-up visits. By focusing on patients who are most likely to benefit from such testing, healthcare resources can be utilized more efficiently, reducing unnecessary costs and the environmental burden associated with broader testing protocols. Eventually, such a tailored approach would address barriers to incorporating genetic testing into the routine management of DCM.^35,36^ Additionally, the environmental impact of cardiology procedures, particularly diagnostic imaging, underscores the need for sustainable medical practices. Emphasizing remote consultations and monitoring, reducing the frequency of diagnostic imaging where appropriate, and considering the full lifecycle impact of medical procedures can help minimize the carbon footprint associated with DCM management.^37^ To enhance these strategies, ongoing research is essential. It should focus on understanding the cost–benefit ratios and environmental impacts of current diagnostic and therapeutic schemes, ensuring that management practices for DCM are both economically viable and environmentally responsible.

## Limitations

Several limitations warrant consideration due to their potential for introducing bias. Our systematic review was confined to scientific literature published in English. We reduced the impact of this restriction on our findings by adhering to a rigorous systematic methodology (PRISMA guidelines). Specific recommendations regarding the management of comorbidities associated with DCM were not evaluated, as this falls outside the scope of our review. We acknowledge that scientific statements often rely more heavily on consensus than on rigorous evidence and may not undergo the same development process as formal guidelines; they nonetheless may reflect the collective expertise of clinicians with an interest in the areas of interest, especially where for all guidelines may be limited. Also, we did not evaluate the validity of individual recommendations presented in the guidelines and scientific documents. Instead, we ensured that all included documents were thoroughly developed, as evidenced by their AGREE II scores.

## Conclusions

There is consensus amongst the clinical practice guidelines and scientific statements regarding recommendations for diagnostic definition and first-line diagnostic methods for DCM. Agreement is also found for genetic counselling, indications for targeted endomyocardial biopsy, and the management of advanced HF, with MCS and cardiac transplantation. There are variations in the recommendations for SCD risk stratification and prophylactic ICD implantation, as well as for cascade genetic testing and follow-up for relatives and the role of CT and nuclear imaging. Further research may aid in the characterization of DCM clinical course, both in its genetic and non-genetic forms, definition of the genetic testing for VUSs, prophylactic therapies to prevent DCM onset on genotype-positive carriers, and aetiology-oriented therapeutic approaches.

## Supplementary Material

qcae109_Supplemental_File

## Data Availability

The data underlying this article are available in the article, and in its online supplementary material. Any other data can be made available on reasonable request to the corresponding author.

## References

[1] Arbelo E, Protonotarios A, Gimeno JR, Arbustini E, Barriales-Villa R, Basso C et al. 2023 ESC guidelines for the management of cardiomyopathies. Eur Heart J 2023;44:3503–3626. 37622657 10.1093/eurheartj/ehad194

[2] Rakar S, Sinagra G, Di Lenarda A, Poletti A, Bussani R, Silvestri F et al. Epidemiology of dilated cardiomyopathy. A prospective post-mortem study of 5252 necropsies. The Heart Muscle Disease Study Group. Eur Heart J 1997;18:117–123. 9049523 10.1093/oxfordjournals.eurheartj.a015092

[3] McKenna WJ, Judge DP. Epidemiology of the inherited cardiomyopathies. Nat Rev Cardiol 2021;18:22–36. 32895535 10.1038/s41569-020-0428-2

[4] Gigli M, Merlo M, Graw SL, Barbati G, Rowland TJ, Slavov DB et al. Genetic risk of arrhythmic phenotypes in patients with dilated cardiomyopathy. J Am Coll Cardiol 2019;74:1480–1490. 31514951 10.1016/j.jacc.2019.06.072 PMC6750731

[5] Eldemire R, Mestroni L, Taylor MRG. Genetics of dilated cardiomyopathy. Annu Rev Med 2024;75:417–426. 37788487 10.1146/annurev-med-052422-020535 PMC10842880

[6] Escobar-Lopez L, Ochoa JP, Mirelis JG, Espinosa MÁ, Navarro M, Gallego-Delgado M et al. Association of genetic variants with outcomes in patients with nonischemic dilated cardiomyopathy. J Am Coll Cardiol 2021;78:1682–1699. 34674813 10.1016/j.jacc.2021.08.039

[7] Pinto YM, Elliott PM, Arbustini E, Adler Y, Anastasakis A, Böhm M et al. Proposal for a revised definition of dilated cardiomyopathy, hypokinetic non-dilated cardiomyopathy, and its implications for clinical practice: a position statement of the ESC working group on myocardial and pericardial diseases. Eur Heart J 2016;37:1850–1858. 26792875 10.1093/eurheartj/ehv727

[8] Antonopoulos AS, Xintarakou A, Protonotarios A, Lazaros G, Miliou A, Tsioufis K et al. Imagenetics for precision medicine in dilated cardiomyopathy. Circ Genom Precis Med 2024;17:e004301. 38415367 10.1161/CIRCGEN.123.004301

[9] Moher D, Liberati A, Tetzlaff J, Altman DG, Group PRISMA. Preferred reporting items for systematic reviews and meta-analyses: the PRISMA statement. PLoS Med 2009;6:e1000097. 19621072 10.1371/journal.pmed.1000097 PMC2707599

[10] Bozkurt B, Colvin M, Cook J, Cooper LT, Deswal A, Fonarow GC. Current diagnostic and treatment strategies for specific dilated cardiomyopathies: a scientific statement from the American Heart Association. Circulation 2016;134:e579–e646. 27832612 10.1161/CIR.0000000000000455

[11] Kitaoka H, Tsutsui H, Kubo T, Ide T, Chikamori T, Fukuda K et al. JCS/JHFS 2018 guideline on the diagnosis and treatment of cardiomyopathies. Circ J 2021;85:1590–1689. 34305070 10.1253/circj.CJ-20-0910

[12] Hershberger RE, Givertz MM, Ho CY, Judge DP, Kantor PF, McBride KL et al. Genetic evaluation of cardiomyopathy—a Heart Failure Society of America Practice Guideline. J. Card Fail. 2018;24:281–302. 29567486 10.1016/j.cardfail.2018.03.004 PMC9903357

[13] Wahbi K, Ben Yaou R, Gandjbakhch E, Anselme F, Gossios T, Lakdawala NK et al. Development and validation of a new risk prediction score for life-threatening ventricular tachyarrhythmias in laminopathies. Circulation 2019;140:293–302. 31155932 10.1161/CIRCULATIONAHA.118.039410

[14] Verstraelen TE, van Lint FHM, Bosman LP, de Brouwer R, Proost VM, Abeln BGS et al. Prediction of ventricular arrhythmia in phospholamban p.Arg14del mutation carriers-reaching the frontiers of individual risk prediction. Eur Heart J 2021;42:2842–2850. 34113975 10.1093/eurheartj/ehab294 PMC8325776

[15] Owen R, Buchan R, Frenneaux M, Jarman JWE, Baruah R, Lota AS et al. Sex differences in the clinical presentation and natural history of dilated cardiomyopathy. JACC Heart Fail 2024;12:352–363. 38032570 10.1016/j.jchf.2023.10.009 PMC10857810

[16] de Frutos F, Ochoa JP, Navarro-Peñalver M, Baas A, Bjerre JV, Zorio E et al. Natural history of MYH7-related dilated cardiomyopathy. J Am Coll Cardiol 2022;80:1447–1461. 36007715 10.1016/j.jacc.2022.07.023

[17] Merlo M, Pivetta A, Pinamonti B, Stolfo D, Zecchin M, Barbati G et al. Long-term prognostic impact of therapeutic strategies in patients with idiopathic dilated cardiomyopathy: changing mortality over the last 30 years. Eur J Heart Fail 2014;16:317–324. 24464640 10.1002/ejhf.16

[18] Merlo M, Pyxaras SA, Pinamonti B, Barbati G, Di Lenarda A, Sinagra G. Prevalence and prognostic significance of left ventricular reverse remodeling in dilated cardiomyopathy receiving tailored medical treatment. J Am Coll Cardiol 2011;57:1468–1476. 21435516 10.1016/j.jacc.2010.11.030

[19] Asselbergs FW, Sammani A, Elliott P, Gimeno JR, Tavazzi L, Tendera M et al. Differences between familial and sporadic dilated cardiomyopathy: ESC EORP cardiomyopathy & myocarditis registry. ESC Heart Fail 2021;8:95–105. 33179448 10.1002/ehf2.13100 PMC7835585

[20] Charron P, Elliott PM, Gimeno JR, Caforio ALP, Kaski JP, Tavazzi L et al. The Cardiomyopathy Registry of the EURObservational Research Programme of the European Society of Cardiology: baseline data and contemporary management of adult patients with cardiomyopathies. Eur Heart J 2018;39:1784–1793. 29378019 10.1093/eurheartj/ehx819

[21] Xu Y, Li W, Wan K, Liang Y, Jiang X, Wang J et al. Myocardial tissue reverse remodeling after guideline-directed medical therapy in idiopathic dilated cardiomyopathy. Circ Heart Fail 2021;14:e007944. 33185117 10.1161/CIRCHEARTFAILURE.120.007944

[22] Selvanayagam JB, Hartshorne T, Billot L, Grover S, Hillis GS, Jung W et al. Cardiovascular magnetic resonance-GUIDEd management of mild to moderate left ventricular systolic dysfunction (CMR GUIDE): study protocol for a randomized controlled trial. Ann Noninvasive Electrocardiol 2017;22:e12420. 28117536 10.1111/anec.12420 PMC6931571

[23] Gatzoulis KA, Dilaveris P, Arsenos P, Tsiachris D, Antoniou CK, Sideris S et al. Arrhythmic risk stratification in nonischemic dilated cardiomyopathy: the ReCONSIDER study design—a two-step, multifactorial, electrophysiology-inclusive approach. Hellenic J Cardiol 2021;62:169–172. 32330568 10.1016/j.hjc.2020.03.008

[24] Flett A, Cebula A, Nicholas Z, Adam R, Ewings S, Prasad S et al. Rationale and study protocol for the BRITISH randomized trial (Using cardiovascular magnetic resonance identified scar as the benchmark risk indication tool for implantable cardioverter defibrillators in patients with nonischemic cardiomyopathy and severe systolic heart failure). Am Heart J 2023;266:149–158. 37777041 10.1016/j.ahj.2023.09.008

[25] Asatryan B, Murray B, Tadros R, Rieder M, Shah RA, Sharaf Dabbagh G et al. Promise and peril of a genotype-first approach to mendelian cardiovascular disease. J Am Heart Assoc 2024;13:e033557. 39424414 10.1161/JAHA.123.033557 PMC11935662

[26] Wilde AAM, Semsarian C, Márquez MF, Shamloo AS, Ackerman MJ, Ashley EA et al. European Heart Rhythm Association (EHRA)/Heart Rhythm Society (HRS)/Asia Pacific Heart Rhythm Society (APHRS)/Latin American Heart Rhythm Society (LAHRS) Expert Consensus statement on the state of genetic testing for cardiac diseases. Europace 2022;24:1307–1367. 35373836 10.1093/europace/euac030 PMC9435643

[27] Chahal CAA, Landstrom AP. Predicting the development of dilated cardiomyopathy in kindred with genetic risk: family matters. J Am Coll Cardiol 2024;83:1652–1655. 38658104 10.1016/j.jacc.2024.03.381

[28] Kurzlechner LM, Kishnani S, Chowdhury S, Atkins SL, Moya-Mendez ME, Parker LE et al. *DiscoVari* : a web-based precision medicine tool for predicting variant pathogenicity in cardiomyopathy- and channelopathy-associated genes. Circ Genom Precis Med 2023;16:317–327. 37409478 10.1161/CIRCGEN.122.003911 PMC10527712

[29] Stroeks SLVM, Hellebrekers DMEI, Claes GRF, Tayal U, Krapels IPC, Vanhoutte EK et al. Clinical impact of re-evaluating genes and variants implicated in dilated cardiomyopathy. Genet Med 2021;23:2186–2193. 34194005 10.1038/s41436-021-01255-1 PMC7614766

[30] Ricci F, Banihashemi B, Pirouzifard M, Sundquist J, Sundquist K, Sutton R et al. Familial risk of dilated and hypertrophic cardiomyopathy: a national family study in Sweden. ESC Heart Fail 2023;10:121–132. 36169166 10.1002/ehf2.14171 PMC9871695

[31] Pepin ME, Ha CM, Crossman DK, Litovsky SH, Varambally S, Barchue JP et al. Genome-wide DNA methylation encodes cardiac transcriptional reprogramming in human ischemic heart failure. Lab Invest 2019;99:371–386. 30089854 10.1038/s41374-018-0104-x PMC6515060

[32] Køber L, Thune JJ, Nielsen JC, Haarbo J, Videbæk L, Korup E et al. Defibrillator implantation in patients with nonischemic systolic heart failure. N Engl J Med 2016;375:1221–1230. 27571011 10.1056/NEJMoa1608029

[33] Marijon E, Narayanan K, Smith K, Barra S, Basso C, Blom MT et al. The Lancet Commission to reduce the global burden of sudden cardiac death: a call for multidisciplinary action. Lancet 2023;402:883–936. 37647926 10.1016/S0140-6736(23)00875-9

[34] Grote Beverborg N, Später D, Knöll R, Hidalgo A, Yeh ST, Elbeck Z et al. Phospholamban antisense oligonucleotides improve cardiac function in murine cardiomyopathy. Nat Commun 2021;12:5180. 34462437 10.1038/s41467-021-25439-0 PMC8405807

[35] Cannatà A, Merlo M, Dal Ferro M, Barbati G, Manca P, Paldino A et al. Association of titin variations with late-onset dilated cardiomyopathy. JAMA Cardiol 2022;7:371. 35138330 10.1001/jamacardio.2021.5890 PMC8829739

[36] Escobar-Lopez L, Ochoa JP, Royuela A, Verdonschot JAJ, Dal Ferro M, Espinosa MA et al. Clinical risk score to predict pathogenic genotypes in patients with dilated cardiomyopathy. J Am Coll Cardiol 2022;80:1115–1126. 36109106 10.1016/j.jacc.2022.06.040 PMC10804447

[37] Khanji MY, Patel R, Ricci F. Going green in cardiology. Eur Heart J 2024;45:411–412. 37889072 10.1093/eurheartj/ehad703

